# The Lesion Known as "Charcot's Joint"

**Published:** 1909-11-27

**Authors:** 


### Surgery.

### THE LESION KNOWN AS "CHARCOT'S JOINT."

Of all surgical lesions few are more difficult to diagnose and treat correctly than those which occur in connection with joints, and of these again the most perplexing are those which are classed together under the term "neuropathic arthritis." The commonest of these is found in association with tabes dorsalis, and the connection between the two was first pointed out by the late Professor Charcot.

The difficulty in the diagnosis is due essentially to the fact that the changes in the joint do not conform to any definite type, and also because the condition is generally seen in its early stages when it presents no single point of resemblance to the descriptions and illustrations of the affection commonly given, in which the normal outline of the joint is entirely lost and knobs and excrescences, due to overgrowth of the ends of the bones, are visible.

### A Typical History.

What occurs in the early stages is a sudden distension of the joint with fluid as the result of an accident which in itself is quite slight. The following history is a more or less typical one. A workman, aged 45, engaged in building operations, slipped upon some scaffolding, but recovered himself without actually falling. He thought he had slightly strained his left knee, but he felt no pain in it, and he continued his work for the rest of the day, a matter of two or three hours. As soon as he arrived home, however, the knee began to swell, and when he was seen the next morning there was an enormous fusiform swelling of the left knee-joint. It was so tense that it gave the impression of being hard, and there were dilated veins upon its surface. Seeing it then for the first time and not knowing the history of its sudden onset, one would undoubtedly have made a provisional diagnosis of sarcoma of the lower end of the femur. The swelling was quite painless, and on careful palpation it could be felt to be a tense cystic swelling rather than a solid one. The patient did not remember ever to have been ill before, and he denied having had syphilis. He said that if he had had it he had been unaware of it, and he had certainly not been treated for it. But examination revealed that he was then suffering from tabes dorsalis; Romberg's sign was well marked and there was a typical Argyll-Robertson pupil. He was put to bed and the limb kept at rest on a splint, but as the effusion showed no tendencies to disappear the joint was aspirated and an elastic bandage applied to the knee. In spite of this the fluid reaccumulated several times, and three aspirations were necessary in all. The joint was, of course, much weakened owing to the stretching of the periarticular structures, but he had a moderately useful limb.

This history is a more or less typical one in early cases of Charcot's joint. It is difficult to understand the suddenness of the effusion following on a slight accident. It is to be presumed that there is some syphilitic disease of the synovial membrane by which the equilibrium of its lymphatic supply is impaired, as is the case in the tunica vaginalis testis when a hydrocele is present, but it must also be supposed that the capsule and periarticular structures are already weakened by disease before the effusion appears, and that they are thus unable to resist any pressure brought to bear upon them.

### Prognosis.

The prognosis is not good. Although a temporary cure may be obtained, as in the case recorded above, the condition tends to recur, and each recurrence leaves the joint weaker than before so that it eventually becomes incapable of performing its functions, and a weak, flail-like joint results.

In the later stages secondary changes occur in the articular ends of the bones entering into the formation of the joint, changes diametrically opposed to each other, in one case hypertrophy and in the other atrophy.

Atrophic changes are more usually found in the class of case described above in which there is great effusion; the hypertrophic variety is generally found when the amount of fluid is small. The hypertrophy consists in the outgrowth of a number of osteophytic processes from the articular end of the bone at the edge of the cartilage. It will thus be seen that the condition presents many points of apparent similarity to osteoarthritis. But the differential diagnosis is readily made if one bears in mind the *rapid onset* and the *painlessness* of a tabetic joint. The discovery of evidence of tabes, of course, clinches the question.

### Treatment.

The treatment is a matter of the greatest difficulty. In the early stages the first object is to get rid of the fluid. The patient should be put to bed and the affected limb kept upon a splint. If the effusion does not subside spontaneously, aspiration must be performed and a firm bandage applied to the limb—an elastic one is best. Even then reaccumulation is likely to occur, and repeated tappings may be necessary.

In the later stages the trouble that has to be overcome is the loss of stability in the joint owing to the laxness of the surrounding structures, which have been stretched beyond their powers of repair. In some cases a surgical instrument may be devised which will restore the function of the limb, but in others the joint is paralysed and flail-like.

This is less inconvenient in the joints of the arm, where increased mobility is not a matter of great moment. But in the case of the knee or ankle it prevents the patient from getting about. For these conditions arthrodesis has been advocated—that is, practically an excision of the joint, with subsequent bony union. The bones unite fairly well after this operation, and in some cases a successful result is obtained; but if it fails amputation is the only alternative.

